# Efficacy of aripiprazole long‐acting injection monotherapy in pregnancy‐related hypomania: A treatment choice through shared decision‐making

**DOI:** 10.1002/pcn5.70225

**Published:** 2025-10-12

**Authors:** Yoshiyo Oguchi, Atsuo Nakagawa, Hiroki Kocha

**Affiliations:** ^1^ Department of Neuropsychiatry St. Marianna University School of Medicine Miyamae‐ku Kawasaki Japan

**Keywords:** aripiprazole long‐acting injection, bipolar disorder, hypomania, measurement‐based care, pregnancy, shared decision‐making

## Abstract

**Background:**

Managing bipolar disorder during pregnancy requires balancing maternal mental health and fetal safety. While guidelines often focus on severe mania, the management of hypomania, a common clinical challenge, remains poorly defined. Although shared decision‐making (SDM) is recommended for treatment planning, its application in managing acute mood episodes during pregnancy remains underexplored, especially without robust evidence.

**Case Presentation:**

A woman in her thirties with DSM‐5 Bipolar I Disorder developed a hypomanic episode at 32 weeks of gestation while on aripiprazole once‐monthly (AOM) 400 mg monotherapy. Although antipsychotic augmentation was considered, limited evidence and strong patient and family concerns regarding fetal exposure led to an SDM‐based approach. The AOM monotherapy was continued with intensive monitoring. Hypomanic symptoms resolved within 4 weeks (Young Mania Rating Scale scores decreased from 14 to 1), with corresponding improvements in functional scores (Global Assessment of Functioning and the Social and Occupational Functioning Assessment Scale), and the patient delivered a healthy infant at term. She remained euthymic at 7 months postpartum.

**Conclusion:**

SDM, when combined with intensive measurement‐based care, may effectively manage acute hypomanic episodes during pregnancy. This case demonstrates that a conservative approach, guided by SDM, can be a viable strategy for managing pregnancy‐related hypomania, highlighting the importance of an individualized treatment plan that respects patient values in a clinical setting with limited evidence.

## BACKGROUND

Bipolar disorder affects approximately 1%–2% of women of childbearing age; moreover, pregnancy is a period of heightened vulnerability to mood destabilization.^1^ It is estimated that 25%–30% of women with bipolar disorder experience mood episodes during pregnancy^2^; however, most research and clinical guidelines focus on severe mania management, offering limited guidance for hypomania.

Shared decision‐making (SDM) is a collaborative process in which clinicians and patients jointly participate in making health decisions after discussing treatment options and considering available evidence, risks, benefits, and patient values.^3^ SDM has been associated with improved treatment adherence and satisfaction,^4^ and its implementation is officially recommended in Japanese perinatal mental health guidelines.^5^ Nonetheless, its role in managing acute episodes during pregnancy, especially when evidence is limited, remains largely unexplored.

We present a case demonstrating the effective use of SDM in the acute management of pregnancy‐related hypomania, which was achieved without pharmacological augmentation and supported by intensive monitoring.

## CASE PRESENTATION

A woman in her thirties with DSM‐5 Bipolar I Disorder was receiving outpatient follow‐up after a manic episode requiring hospitalization in October, Year X. Given her desire to conceive in her thirties and a history of treatment with mood stabilizers with teratogenic potential (lithium and valproate), a preconception consultation was conducted involving her psychiatrist and a pharmacist. Her regimen was transitioned from mood stabilizers to oral aripiprazole 24 mg daily. After confirming its efficacy and tolerability, she was switched to aripiprazole once‐monthly (AOM) 400 mg in January of Year X + 1 to ensure adherence and prevent relapse, achieving stable euthymia before conception. This plan was shared with her obstetrician, who agreed with the approach. She was counseled to maintain monthly psychiatric follow‐ups and long‐acting injectable (LAI) administration throughout the perinatal period, and to immediately report any obstetric or psychiatric concerns. She became pregnant in March of Year X + 1 and remained clinically stable during the first and second trimesters while on AOM treatment.

At 32 weeks of gestation, she developed the following hypomanic symptoms: decreased need for sleep (3–4 h/night), simultaneous engagement in multiple tasks, pressured speech, racing thoughts, and elevated mood. The Young Mania Rating Scale (YMRS) score was 14. She retained full insight and expressed concerns regarding the potential impact of her symptoms on the pregnancy. No triggers were identified. The thyroid and other laboratory evaluations were unremarkable.

The clinical team considered pharmacological augmentation with low‐dose olanzapine or quetiapine for rapid stabilization. Nevertheless, given the lack of studies addressing hypomania management during pregnancy and the patient and her family's strong concerns about fetal medication exposure, we adopted an SDM approach.

Using a structured SDM process, we presented three options: (1) continued AOM monotherapy with intensive monitoring; (2) addition of low‐dose olanzapine (2.5 mg); and (3) addition of quetiapine (25–50 mg). Each approach was discussed in terms of symptom resolution, sleep improvement, safety profiles, potential metabolic effects, and potential fetal impact. The patient prioritized minimal pharmacological exposure and expressed a willingness to accept slower symptom improvement in exchange for reducing the theoretical risk to the fetus.

A consensus was reached to continue AOM monotherapy, accompanied by a detailed monitoring plan: weekly psychiatric evaluation with standardized scales, regular mood charting by the patient, emergency contact access, and a prearranged crisis plan. Her husband, who also attended the consultation, agreed to maintain a daily log of observed behaviors and sleep patterns and to accompany her to all appointments.

**Figure 1:**
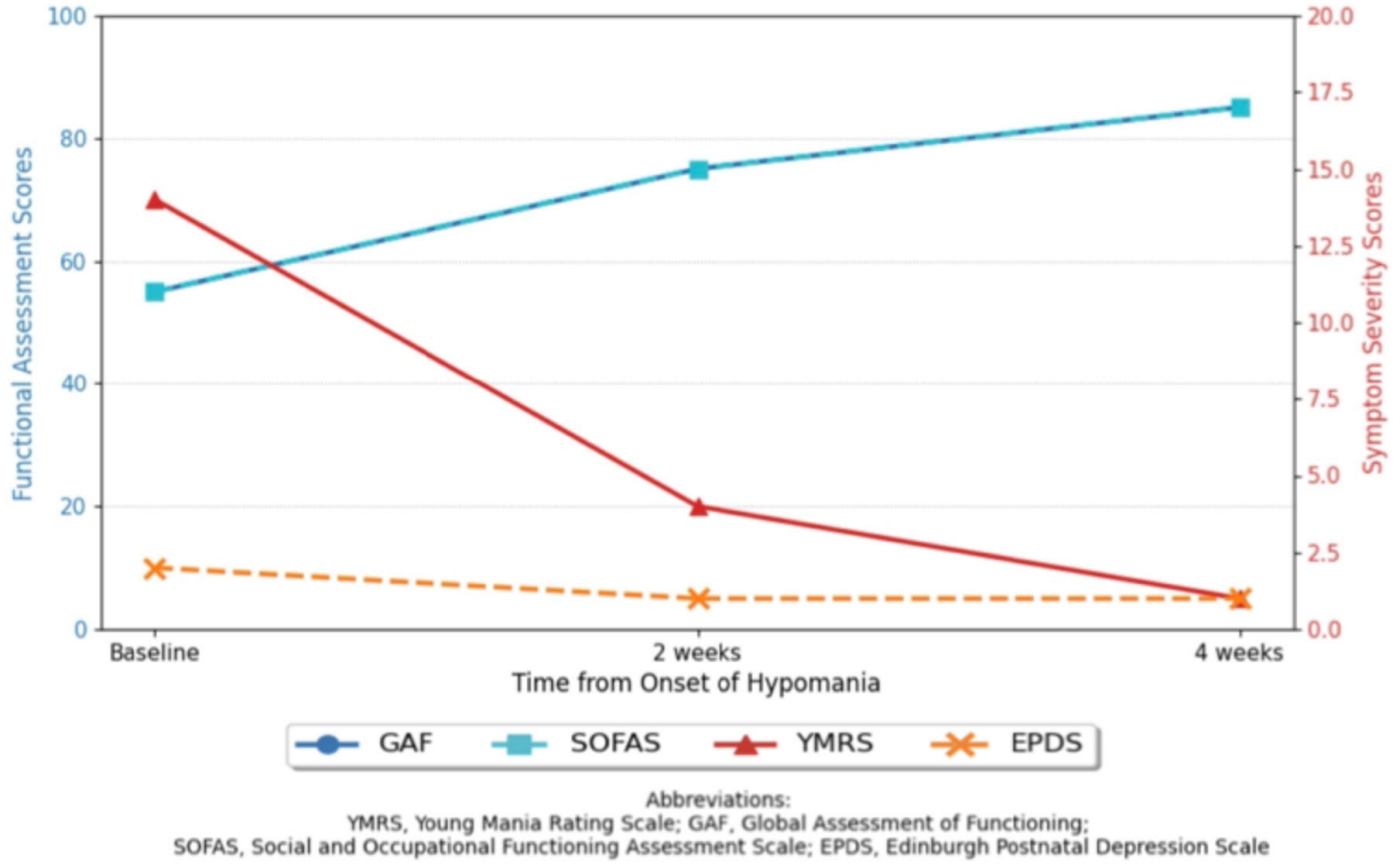
Changes in clinical and functional scores over time. The graph illustrates the patient's clinical and functional progress from the onset of hypomania at 32 weeks of gestation. The primary *y*‐axis (left, blue) shows scores for the Global Assessment of Functioning (GAF) and Social and Occupational Functioning Assessment Scale (SOFAS). The secondary *y*‐axis (right, red) shows scores for the Young Mania Rating Scale (YMRS) and the Edinburgh Postnatal Depression Scale (EPDS). Over 4 weeks of continued aripiprazole once‐monthly (AOM) monotherapy with intensive monitoring, YMRS scores decreased, while GAF and SOFAS scores improved, indicating both symptom resolution and functional recovery. EPDS scores remained low throughout.

Her clinical course was closely monitored using multiple assessment tools (Figure 1). Hypomanic symptoms improved, with YMRS scores declining from 14 at baseline to 4 at 2 weeks and 1 at 4 weeks. Functional improvement was also observed, with the Global Assessment of Functioning (GAF) score increasing from 55 to 85 and the Social and Occupational Functioning Assessment Scale (SOFAS) score increasing from 55 to 85 over the same period. Depressive symptoms remained minimal, with Edinburgh Postnatal Depression Scale (EPDS) scores consistently low. The patient delivered a healthy male infant at 39 weeks of gestation without perinatal complications. No signs of poor neonatal adaptation syndrome were observed. Seven months postpartum, the patient remained euthymic on AOM monotherapy.

## DISCUSSION

This case highlights the novelty and effectiveness of applying an SDM framework to the conservative management of an acute hypomanic episode during late pregnancy—a clinical scenario for which clear evidence‐based guidelines are lacking. Because current guidelines predominantly address severe manic or depressive episodes, they leave clinicians with little guidance for managing hypomania.^2^, ^6^ Therefore, individualized approaches grounded in patient values and supported by rigorous monitoring are warranted. The novelty of this case lies in the application of SDM, traditionally used for treatment initiation, to the management of acute episodes during pregnancy.

The decision to continue AOM monotherapy was based on a collaborative risk‐benefit analysis. Aripiprazole LAI offers distinct advantages in pregnancy, including stable plasma concentrations that may reduce peak‐level fetal exposure compared to oral antipsychotics,^7^ and assured medication adherence, which is critical for preventing relapse. The safety profile of aripiprazole in pregnancy, while not definitive, is relatively reassuring. A notable case report by Ballester‐Gracia et al.,^8^ a recent scoping review by Teodorescu et al.,^9^ and other case series^10^ suggest that in utero exposure to aripiprazole is not associated with a significant increase in the risk of major congenital malformations. This information was crucial in reassuring the patient and her family, allowing them to participate meaningfully in the decision‐making process.

The management of hypomania during pregnancy requires consideration of various therapeutic options. Mood stabilizers like lithium and lamotrigine are used, but lithium carries a known risk of cardiac anomalies, and lamotrigine levels can fluctuate significantly during pregnancy, complicating its management.^11^, ^12^ Atypical antipsychotics such as olanzapine and quetiapine are also options and have shown efficacy; however, they carry risks of maternal metabolic side effects like gestational diabetes and excessive weight gain.^11^, ^12^ This contrasts with the more favorable metabolic profile of aripiprazole.^13^ Given that this patient's symptoms were of hypomanic severity and she had full insight, the SDM process allowed for a personalized approach that prioritized her desire of minimizing fetal exposure to additional medications, leading to the conservative management strategy.

The success of this approach was contingent upon several key elements integrated into our SDM framework. First, we implemented intensive measurement‐based care, using not only the YMRS for manic symptoms but also the GAF, SOFAS, and EPDS to provide a holistic view of the patient's functional and emotional state. This objective tracking enabled data‐driven adjustments and provided reassurance that the conservative strategy was effective, allowing for prompt intervention if deterioration occurred. Second, a comprehensive crisis plan was established in advance, which reduced anxiety for the patient and family. Third, the active involvement of the patient's husband in daily monitoring created a robust support system, enhancing safety and facilitating early detection of any changes. These elements are broadly applicable and can be implemented in many perinatal psychiatric settings. As this was a single case, validation with larger samples is required. Moreover, identifying which patient characteristics (degree of insight, family support, symptom severity) predict successful conservative management through SDM is necessary. As highlighted by Teodorescu et al.,^9^ more robust research is imperative to provide definitive guidance for managing bipolar disorder with LAI antipsychotics in pregnant patients.

## CONCLUSION

SDM, when integrated with intensive measurement‐based care, can be a cornerstone in managing acute hypomanic episodes during pregnancy, particularly in the absence of high‐level evidence. Our experience suggests that by respecting patient preferences while ensuring a rigorous safety net, it is possible to achieve favorable maternal and neonatal outcomes without immediate pharmacological escalation. This case extends the application of SDM from long‐term treatment planning to acute perinatal care, offering a patient‐centered and safety‐conscious pathway in a complex clinical scenario.

## AUTHOR CONTRIBUTIONS

Yoshiyo Oguchi conceptualized the case report, collected clinical data, and drafted the manuscript. Atsuo Nakagawa and Hiroki Kocha critically reviewed and revised the manuscript for intellectual content. All authors approved the final version of the manuscript.

## CONFLICT OF INTEREST STATEMENT

The authors declare no conflicts of interest.

## ETHICS APPROVAL STATEMENT

Written informed consent was obtained from the patient for the publication of this case report. This case report was prepared in accordance with the Declaration of Helsinki and CARE guidelines.

## PATIENT CONSENT STATEMENT

N/A.

## CLINICAL TRIAL REGISTRATION

N/A.

## Data Availability

Data sharing is not applicable to this article as no datasets were generated or analyzed during the current study.
